# Decades on emergency decision-making: a bibliometric analysis and literature review

**DOI:** 10.1007/s40747-021-00451-5

**Published:** 2021-07-27

**Authors:** Lin-Xiu Hou, Ling-Xiang Mao, Hu-Chen Liu, Ling Zhang

**Affiliations:** 1 School of Management, Shanghai University, Shanghai, 200444 People’s Republic of China; 2 School of Economics and Management, Tongji University, 1239 Siping Road, Shanghai, 200092 People’s Republic of China; 3 College of Economics and Management, China Jiliang University, Hangzhou, 310018 People’s Republic of China; 4 School of Economics and Management, Anhui Normal University, Wuhu, 241002 People’s Republic of China

**Keywords:** Emergency management, Emergency decision-making, Multiple-criteria decision-making, Literature review, Bibliometric analysis

## Abstract

When an emergency occurs, effective decisions should be made in a limited time to reduce the casualties and economic losses as much as possible. In the past decades, emergency decision-making (EDM) has become a research hotspot and a lot of studies have been conducted for better managing emergency events under tight time constraint. However, there is a lack of a comprehensive bibliometric analysis of the literature on this topic. The objective of this paper is to provide academic community with a complete bibliometric analysis of the EDM researches to generate a global picture of developments, focus areas, and trends in the field. A total of 303 journal publications published between 2010 and 2020 were identified and analyzed using the VOSviewer in regard to cooperation network, co-citation network, and keyword co-occurrence network. The findings indicate that the annual publications in this research field have increased rapidly since 2014. Based on the cooperation network and co-citation network analyses, the most productive and influential countries, institutions, researchers, and their cooperation networks were identified. Using the co-citation network analysis, the landmark articles and the core journals in the EDM area are found out. With the help of the keyword co-occurrence network analysis, research hotspots and development of the EDM domain are determined. According to current trends and blind spots in the literature, possible directions for further investigation are finally suggested for EDM. The literature review results provide valuable information and new insights for both scholars and practitioners to grasp the current situation, hotspots and future research agenda of the EDM field.

## Introduction

During the past decades, different kinds of emergency events occurred frequently around the world, such as the nuclear power plant explosion of Japan in 2011, the Thallium pollution event in China in 2017, the explosion at a chemical plant in China in 2019, and the transmission and infection of novel coronavirus in 2019. On the one hand, such disasters can result in environmental pollution and ecological damages [52, 58, 54]; on the other hand, they tend to cause huge loss of life and property [68, 74]. When a devastating emergency occur, a reasonable rescue plan should be made in a short time to reduce casualties and economic losses [16, 41, 46]. However, how to effectively deal with emergencies is a highly challenging issue worldwide. In the process of emergency decision-making (EDM), it is hard to make a correct decision to handle emergency events because of the complexity and uncertainty of emergency events and strong time constraints [51, 64, 65]. Moreover, emergency decisions may have potentially serious risks since a wrong decision may result in unexpected catastrophic consequences [5, 39, 65]. Therefore, a lot of studies have been performed on emergency management and the EDM problems have attracted more attention from both researchers and practitioners in the past few years [2, 11, 14, 16, 33, 34, 66, 69].

The literature review is a commonly used research method to understand scientific contributions related to a research field and provide guidance for future directions [18]. It allows researchers to expand their bibliographic database of a particular topic and avoid the reinvention of already explained issues and existing solutions [23]. Although EDM has witnessed a rapid development and a large number of articles have been published in the past decades, there has been only a few literature reviews of available researches in this area so far. For example, Zhou et al. [78] first elaborated the concepts and characteristics of EDM for natural disasters and then provided an overview of the EDM of natural disasters from a methodological perspective. Chen et al. [8] reviewed the computation intelligence technologies applied in emergency management and summarized the emergency management systems adopted in diverse industries. Yaghoubi et al. [65] carried out a systematic review study to determine effective determinants and components in the decision-making on hospital emergency evacuation in disasters and emergencies. Bond and Cooper [4] reviewed the literature on recognition-primed decision-making and influences on emergency decisions with reference to an ophthalmic critical incident. Besides, two relevant literature reviews focused on medical decision-making tasks have been carried out in Refs. [40, 43].

The above literature review studies provided valuable insights and research agendas worthy of academic attention. But their research periods are relatively early and there is a lack of a bibliometric analysis of the literature on EDM. Compared to general literature reviews, the bibliometric technique is a useful method to understand the features of a large amount of literature in a certain field, which could be done to quantify cooperation relationships, co-citation similarities, major research themes, and future trends [27]. Therefore, the purpose of this article is to review the EDM literature with the help of bibliometric analyses in a systematic way. A total of 303 articles published between 2010 and 2020 were collected from the Web of Science database. Based on the selected articles related to EDM, main contributions of the current work are listed as follows: (1) The research trends according to the number of articles on EDM are identified. (2) The most productive countries, institutions, and authors, and their cooperation relationships are elucidated by the cooperation network analysis. (3) The most significant articles and journals are determined using the co-citation network analysis. (4) The research hotspots and emerging trends are deduced to encourage further developments in the EDM field and to inform future research.

The remainder of this paper is organized as follows: the next section introduces the research methodology and the literature review process of this study. The subsequent section presents the descriptive results of included studies and the results of the bibliometric analysis with the consideration of the above research objectives. Then the most popular EDM methods used in the literature are analyzed. In the penultimate section, limitations missed by EDM researchers and potential research directions are discussed. Finally, conclusions are given.

## Research methodology

To conduct a bibliometric review on the EDM literature, the Web of Science database was selected for data collection, as it is the largest international database including the most important and high-impact journals [28, 77]. The search query was performed using the terms “emergency management”, “emergency decision-making”, and “emergency AND decision-making” in the *Title, Abstract, and Keywords* fields of the database. In addition, document types were refined to the article and the period of literature retrieval was limited to 2010–2020. Only articles published in international journals and English are considered in the analysis. In the beginning, a total of 8493 articles were identified according to the given search strategy, but not all the retrieved articles were relevant to this study. Thus, a further manual screening process was conducted according to titles, abstracts, and full texts of the candidate articles to eliminate unrelated researches. Finally, 303 publications focusing on EDM were retained for further analysis. The complete records, including author, country, title, abstract, citation record, author affiliation of the selected articles were exported in plain text for bibliometric analysis.

For the bibliographic analysis, the following four parameters have been extracted and analyzed. *Total papers* are the total number of articles in the research domain; total citations are the total number of citations received by the articles; citations per paper refers to the total number of citations divided by the total articles; citations per year denote the average number of citations per year for an article.

For realizing the quantitative analysis and visualization of the selected articles, VOSviewer software was applied to conduct the bibliometric analysis in this study. The VOSviewer is a program for constructing and viewing bibliometric maps. It can construct web links to scientific publications, scientific journals, researchers, research organizations, countries, and keywords [38, 53]. In recent years, the VOSviewer has been widely used for bibliometric analyses of many research domains [32, 36, 51].

## Results and discussion

### Publication trend in the EDM field

**Fig. 1:**
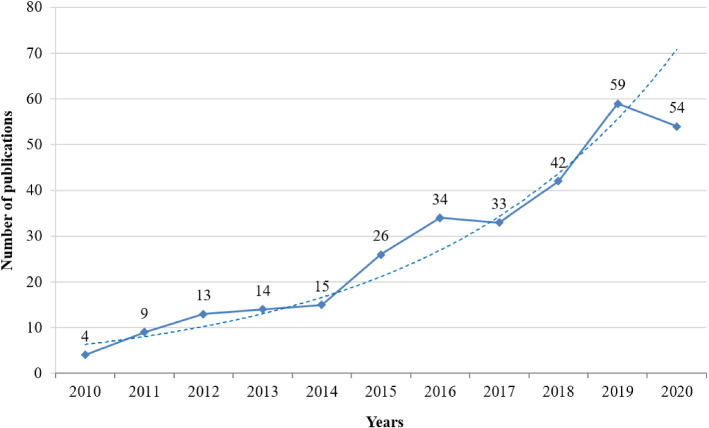
Annual publication quantity trend of EDM

The total number of papers is an important index for measuring the research trend of a research domain. The growth trend of annual publication quantity on EDM is illustrated in Fig. 1. From the figure, it can be seen that the field of EDM is still at an early stage because it was beginning to come into focus since the year 2010 and the total number of publications in 2020 still does not exceed 60. Besides, the EDM studies for handling emergency events have grown significantly during the period of 2010–2020, particularly after 2014. The number of published articles on this theme increased to 59 publications in 2019, from zero paper in 2010; 73.27% of the publications were published in the last five years. This could be due to the fact that various emergencies have occurred more frequently in recent years than ever before and decision-makers increasingly face the need to mitigate the effect of such emergencies on society and the economy. It is forecasted that the number of studies on the topic will continue to increase in the approaching years since how to effectively address EDM problems in time is of great significance to ensure the safety of life and minimize the economic loss of emergencies.

### Cooperation network analysis

#### Distribution of countries

The 303 documents retrieved on EDM are from 37 countries and regions. Table 1 lists the most productive countries according to the total papers of each country. It can be observed that China has the largest number of articles with 126 papers; the USA ranks second with 48 papers. The most noteworthy is that developing countries like China, Iran, and India rank in the top ten countries. This shows that these countries are playing a more and more important part in the EDM field. According to the total citations, China is the country with the most citations and thus has the highest level of influence in the EDM domain. It is followed by the USA, Turkey, England, Spain, and India. Besides, Turkey has the highest number of citations per paper at 47.60, with 5 published papers and 238 citations; USA is next in terms of the citations per paper with a value of 20.88.

**Table 1:** The top 10 productive countries in EDM

Rank	Country	Total papers	Total citations	Citations per paper
1	China	126	1410	11.19
2	USA	48	1002	20.88
3	England	19	231	12.16
4	Australia	15	100	6.67
5	Spain	9	169	18.78
6	India	8	145	18.13
7	Italy	7	111	15.86
8	Canada	6	92	15.33
9	Iran	6	37	6.17
10	Netherlands	6	20	3.33
11	Turkey	5	238	47.60

**Fig. 2:**
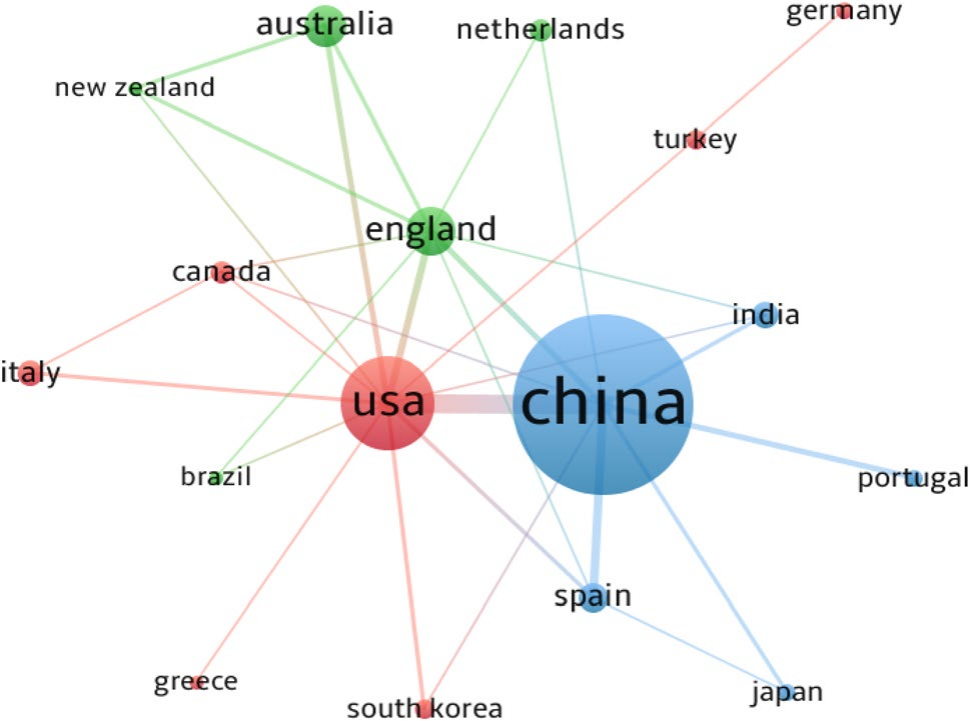
Cooperation network of the most productive countries

Figure 2 presents the cooperation network between the most productive countries. The threshold for the minimum number of documents of a country was set at three. The sizes of the nodes show the number of articles, the links between the nodes represent the collaboration between them, the colors represent their collaboration clusters. It can be seen that China is the most active country and cooperates with other nine countries, especially with the USA, England, and Spain; the USA cooperates with other 12 countries, especially with China. As shown in Fig. 2, China, Japan, Spain, India, and Portugal form a cluster, which indicates that these countries focused on the same research topic. Similarly, England, Brazil, Australia, Netherlands, and New Zealand form a cluster. Therefore, it can be concluded that the cooperation between countries is relatively close in the EDM field. International cooperation between institutions is an effective way to share knowledge, which should be strengthened to advance and promote the EDM development worldwide.

#### Distribution of research institutions

Table 2 lists the top 10 productive institutions in the EDM field according to their total published papers. As we can see, the most productive institution is Wuhan University of Technology, which published 10 articles, followed by Sichuan University (9) and Tongji University (9). As far as the total citations, Sichuan University, Tongji University, and Fuzhou University are ranked in the top three, attracting wide attention from scholars in the field. As for the citations per paper, Nanjing University of Information Science and Technology ranks the highest at 24.2, followed by Sichuan University (23.44) and Fuzhou University (18.25). In addition, Table 2 shows that the top 10 productive institutions are all from China; most of them are dominant according to the total papers and total citations. The results indicate that China and its higher education institutions occupy an overwhelming dominant position in the EDM field.

**Table 2:** The 10 productive institutions in EDM

Rank	Institutions	Country	Total papers	Total citations	Citations per paper
1	Wuhan University of Technology	China	10	65	6.5
2	Sichuan University	China	9	211	23.44
3	Tongji University	China	9	149	16.56
4	Fuzhou University	China	8	146	18.25
5	Central South University	China	7	22	3.14
6	Chinese Academy of Sciences	China	6	18	3.00
7	Tsinghua University	China	6	103	15.00
8	University of Science and Technology of China	China	5	38	17.17
9	Nanjing University of Information Science and Technology	China	5	121	24.2
10	Wuhan University	China	5	19	3.8

**Fig. 3:**
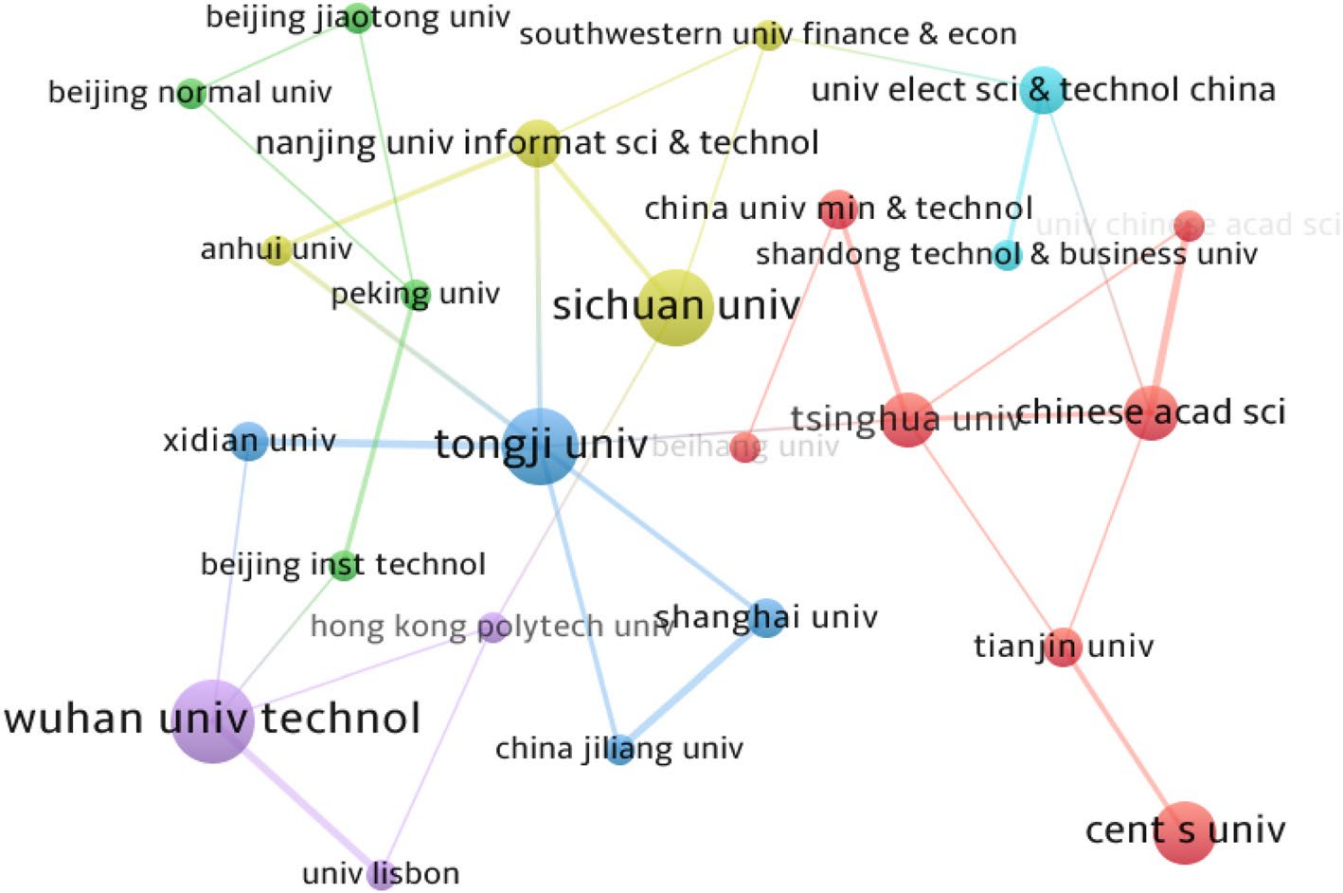
Cooperation network of the most productive institutions

Figure 3 shows the institution cooperation network of the retrieved EDM publications, in which nodes represent institutions, colors express the clusters of institutions, and links denote the strength of collaboration between institutions. In addition, the cores of the network correspond to the most prolific institutions summarized in Table 2. As can be seen from Fig. 3, the nodes of Tongji University, Shanghai University, Xidian University, and China Jiliang University have the same color. This indicates these four institutions have focused on similar research topics. The same is true for Nanjing University of Information Science and Technology, Sichuan University, Southwestern University of Finance and Economics, and Anhui University.

Considering the thickness of the links in Fig. 3, it can be found that the collaboration between Chinese institutions is relatively close. However, cross-regional cooperation between countries is weak, which can be seen from the cooperation among Wuhan University of Technology, the Hong Kong Polytechnic University and the University of Lisbon. In terms of the link number, Tongji University (6 links) has cooperated with others most in the EDM area, followed by Tsinghua University (5 links), Wuhan University of Technology (4 links), Chinese Academy of Sciences (4 links), and Nanjing University of Information Science & Technology (4 links). Here, it is possible to conclude that the most productive institutions are mostly from China, which reflects that China is the main research force of the EDM field.

#### Authors and co-authorship relationship

The most prolific authors and the most reputable research groups in the EDM field can be identified by mapping the author co-authorship network. In total, there are 955 authors from the selected 303 articles on EDM. The most productive and important authors in the EDM field are listed in Table 3 according to the number of publications. From Table 3, it can be seen that the most productive author is Xuanhua Xu with 11 articles, followed by Xiaohong Chen and Zeshui Xu with 9 and 7 publications, respectively. Regarding the total citations, Xuanhua Xu ranks the first with 255 total citations; Xiaohong Chen and Huayou Chen are in the second and the third positions with 249 and 190 citations, respectively. It is worth mentioning that Ligang Zhou has the highest citations per paper although being in the 10th place.

**Table 3:** The top 10 productive authors in EDM

Rank	Author	Total papers	Total citations	Citations per paper
1	Xuanhua Xu	11	255	23.18
2	Xiaohong Chen	9	249	27.67
3	Zeshui Xu	7	118	16.86
4	Yanbing Ju	7	142	20.29
5	Yingming Wang	7	159	22.71
6	Huayou Chen	5	190	38.00
7	Weimin Ma	5	125	25.00
8	Bingzhen Sun	5	125	25.00
9	Aihua Wang	5	122	24.40
10	Ligang Zhang	4	160	40.00

**Fig. 4:**
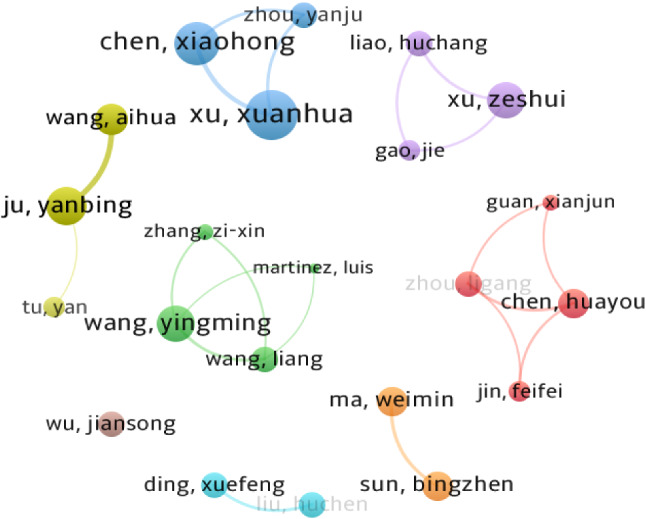
Co-authorship network of authors

Figure 4 shows the co-authorship network of authors in the EDM area, in which nodes represent scholars and links reflects the strength of collaboration between them. As can be seen in Fig. 4, Xuanhua Xu and Xiaohong Chen from Central South University published more cooperative documents in the co-authorship network. They published seven collaborative documents, which is consistent with the previous result that Central South University is a productive institution in EDM. Huayou Chen (Anhui University), Xianjun Guan (Tongji University), Ligang Zhou (Anhui University), and Feifei Jin (Hefei University of Technology) published five cooperative documents. Yingming Wang, who is affiliated with Fuzhou University, published five cooperative articles and is the most prolific scholar in the green cluster. In this cluster, Liang Wang and Zixin Zhang are also from Fuzhou University, while Martinez Luis is from the University of Jaen and has academic collaboration with Wang Liang. It can be concluded that the collaboration between the authors in EDM is relatively weak according to the number of papers. Up to now, several main research teams have been formed, but the connection among them is weak and academic communications are few.

### Citation and co-citation network analysis

#### Document citation and co-citation analysis

Citation analysis is a useful method to assess the impact of an article and find the most important articles in a certain field. Table 4 summarizes the most important publications according to their average citations and total citations. As one can observe, the most influential paper is Ref. [68], which provided a case study to investigate how social media technologies were used, what influences they made on knowledge sharing, reuse, and decision-making, and how knowledge was maintained in these systems. The second most influential article is Ref. [57], which proposed an approach based on the Hamacher aggregation operators under single-valued neutrosophic 2-tuple linguistic environment and applied it to emergency management. The next two influential articles are Ref. [25] and Ref. [56] with their average citations 32.50 and 32.00, respectively. Han and Deng [25] suggested an enhanced fuzzy evidential decision-making trial and evaluation laboratory (DEMATEL) method to identify critical success factors in emergency management. Wu et al. [56] introduced an improved method for handling ships without command by incorporating evidential reasoning and technique for order of preference by similarity to ideal solution (TOPSIS) in the decision-making of emergency responses.

**Table 4:** The top 10 cited documents in EDM

Rank	Author	Citations per year	Total citations
1	Yates and Paquette [68]	48.78	439
2	Wu et al. [56, 57]	36.50	73
3	Han and Deng [25]	32.50	65
4	Wu et al. [56]	32.00	64
5	Peng and Garg [39]	30.50	61
6	Xu et al. [63]	30.00	150
7	Wang et al. [52]	22.67	68
8	Wu et al. [55]	22.00	66
9	Sun et al. [47]	22.00	44
10	Noyan [37]	21.13	169

As far as the total citations, the paper of Ref. [68] has the largest total citation (439 times), which reflects the high influence of the study in the EDM field. The second most frequently cited article is Ref. [37]; this paper put forward a risk-averse two-stage stochastic programming model for disaster management. Xu et al. [63] have the third-largest number of citations. This paper proposed an improved consensus model for large group EDM considering minority opinions and non-cooperative behaviors.

**Fig. 5:**
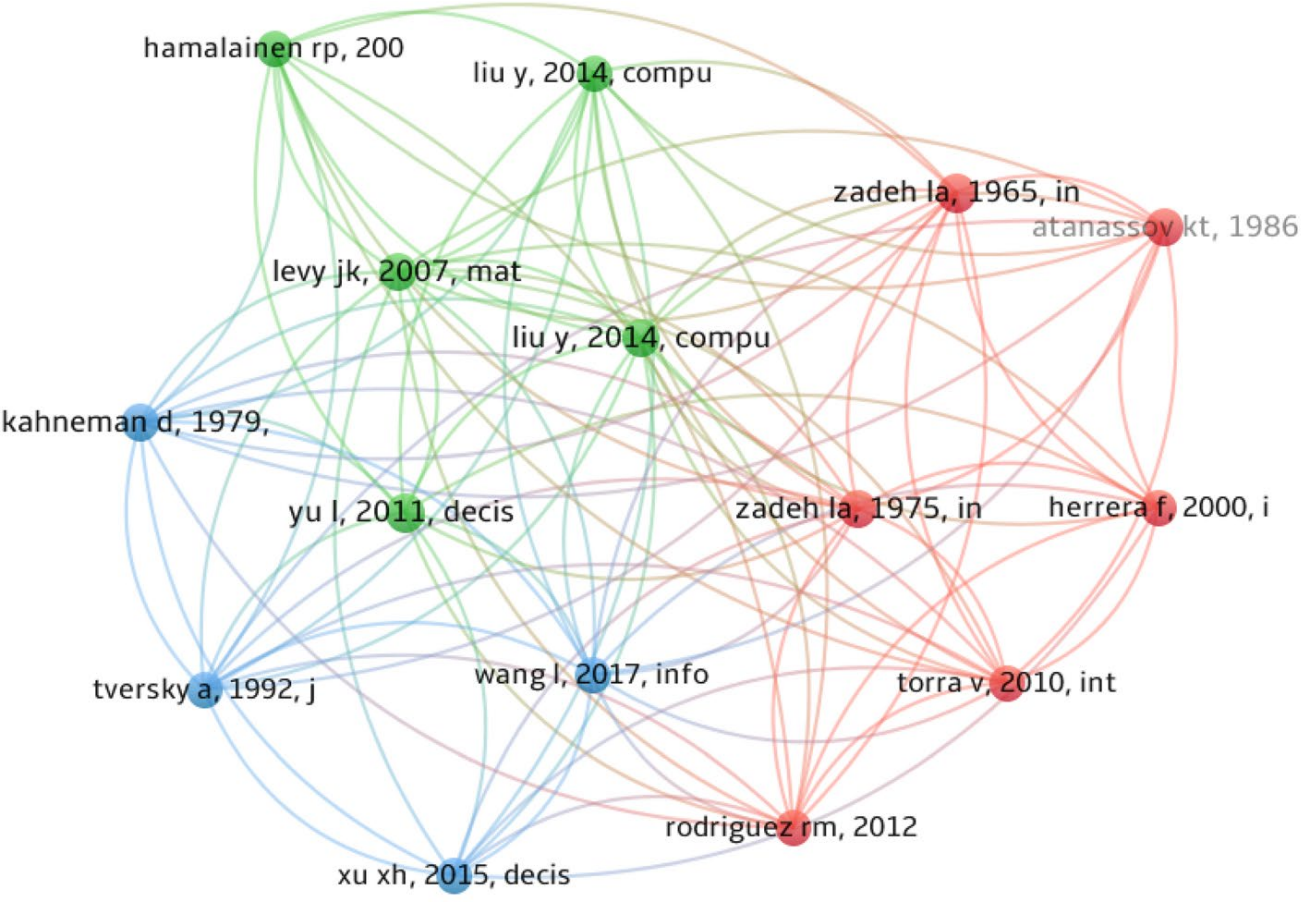
Co-citation network of documents on EDM

Document co-citation analysis is aimed to determine the publications which have a great influence on a research area. In total, there are 9076 references cited by the selected articles in this study. Figure 5 presents the co-citation network of documents being cited more than ten times. In this figure, the sizes of the nodes represent total citations; the thickness of links represents the number of times two publications have been cited together by other articles, and nodes of the common color show the common cluster with similar themes. As shown in Fig. 5, 15 references are segmented into three clusters. The green cluster primarily introduces new EDM methods according to analytic network process (ANP), cumulative prospect theory (CPT), fault tree analysis (FTA) and so on. The paper of [35] has the largest number of links (61) and could be identified as the core of the green cluster. In this paper, a risk decision analysis method based on CPT was proposed to solve the risk decision-making problem in emergency response. The topic of red cluster is fuzzy sets and linguistic variables, in which the paper of [69] is the most influential article and has the largest number of links (42). The concept of linguistic variables and their application to approximate reasoning were described in detail in Ref. [71]. The blue cluster mainly involves the extension and application of prospect theory for EDM, and the article of Ref. [31] has the largest number of links (50), and is the core of this cluster. This article developed the prospect theory to consider experts’ psychological behavior in the decision-making process.

#### Distribution and co-citation analysis of publication sources

Publication sources analysis is helpful to find the core journals of a certain research area, which is important for scholars to search the articles and select an appropriate journal to publish their articles. According to the literature search results, we find that the 303 publications are from 168 publication sources. Table 5 shows the top 10 productive publication sources in the EDM field. It can be seen that International Journal of Disaster Risk Reduction is the most productive publication source with nine articles, followed by Computers Industrial Engineering and IEEE Access with eight articles. As for the total citations, Knowledge-Based Systems receives the most citations of all journals reaching 197 cites; it is followed by Computers Industrial Engineering with 194 cites. As far as the citations per paper, Knowledge-Based Systems ranked first in the listed journals, followed by Computers Industrial Engineering and International Journal of Environmental Research and Public Health.

**Table 5:** The top 10 publication sources in EDM

Rank	Journals	Total papers	Total citations	Citations per paper	Impact factor
1	International Journal of Disaster Risk Reduction	9	59	6.56	2.896
2	Computers Industrial Engineering	8	194	24.25	0.554
3	IEEE Access	8	37	4.63	3.745
4	Knowledge-Based Systems	7	197	28.14	5.921
5	International Journal of Environmental Research and Public Health	7	44	22.00	2.849
6	Sustainability	7	61	8.71	2.576
7	ISPRS International Journal of Geo-Information	6	17	2.83	2.239
8	Journal of Homeland Security and Emergency Management	6	12	2.00	0.394
9	Journal of Intelligent Fuzzy Systems	6	11	1.83	1.637
10	Natural Hazards	6	53	8.83	2.550

**Fig. 6:**
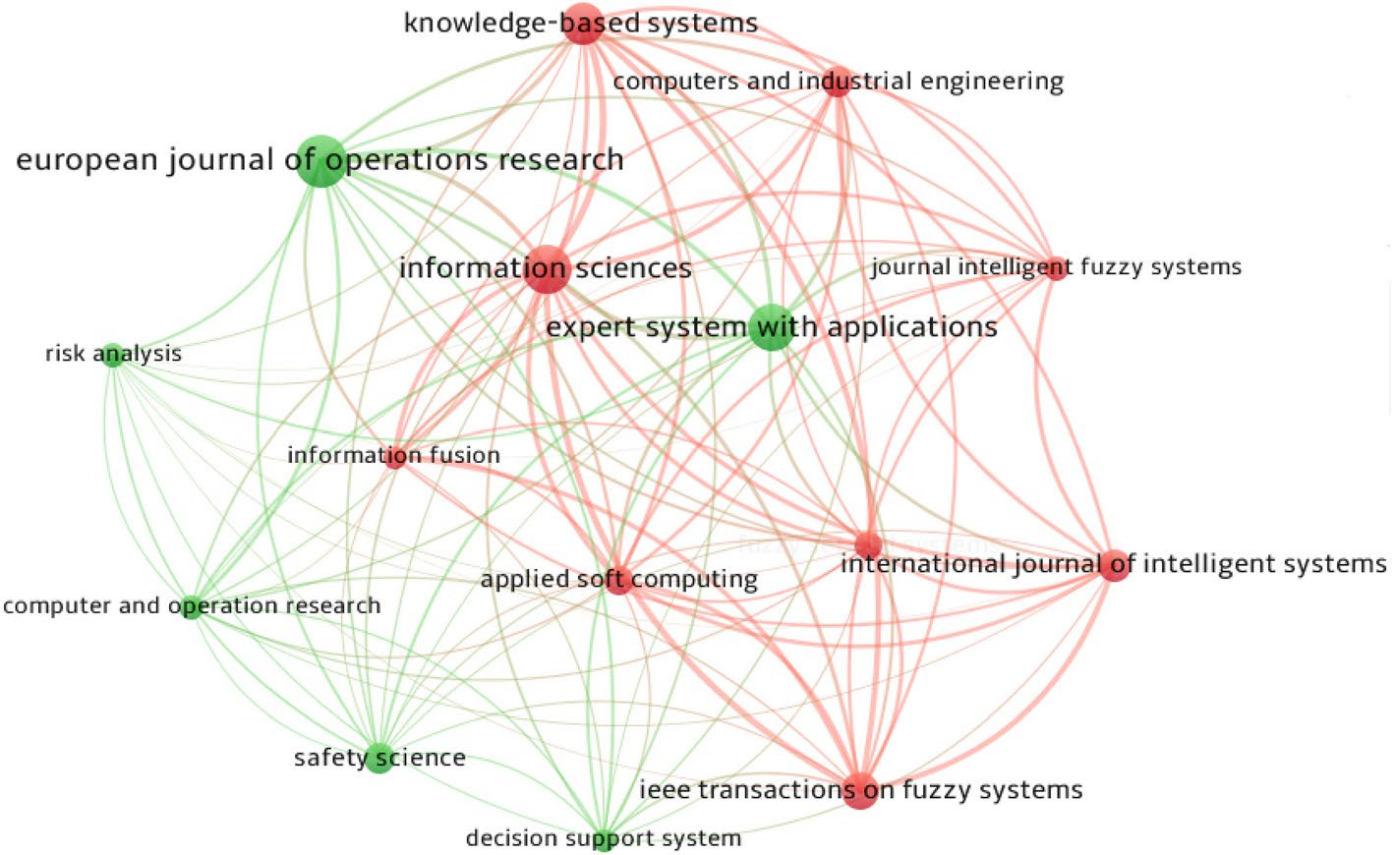
Co-citation network of publication sources in EDM

The purpose of publication sources co-citation analysis is to find the important publication sources most cited in the EDM field. In total, 4268 publication sources are cited by the selected 303 articles. Figure 6 shows the co-citation network of publication sources cited more than 65 times in EDM, where the sizes of nodes represent the number of publication sources citations. As we can see, the node of European Journal of Operational Research is the largest indicating that most of the citations are originated from it. Other important nodes in the EDM area are Expert Systems with Applications, Information Sciences, Knowledge-Based Systems, and IEEE Transactions on Fuzzy Systems. Considering the number of links in the network, Information Sciences ranks first (3810 links) in the red cluster which focuses on risk management techniques and methods. The green cluster focuses on computer science, and European Journal of Operational Research is the core journal with the highest number of links (2520).

### Temporal evolution of hot topics

**Fig. 7:**
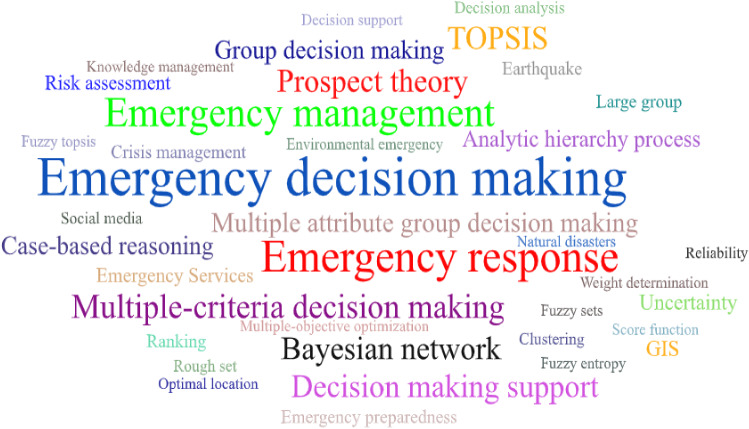
Keyword co-occurrence analysis of the EDM field

Research hotspots and trends related to a certain field can be investigated by the frequency analysis of keywords. Figure 7 shows the keyword co-occurrence network analysis of EDM articles. From this figure, we can see that ‘‘emergency decision-making”, ‘‘emergency response”, “emergency management”, “multiple-criteria decision-making”, “Bayesian network”, “TOPSIS”, “decision-making support”, and “prospect theory” are the keywords taking over notable positions.

**Fig. 8:**
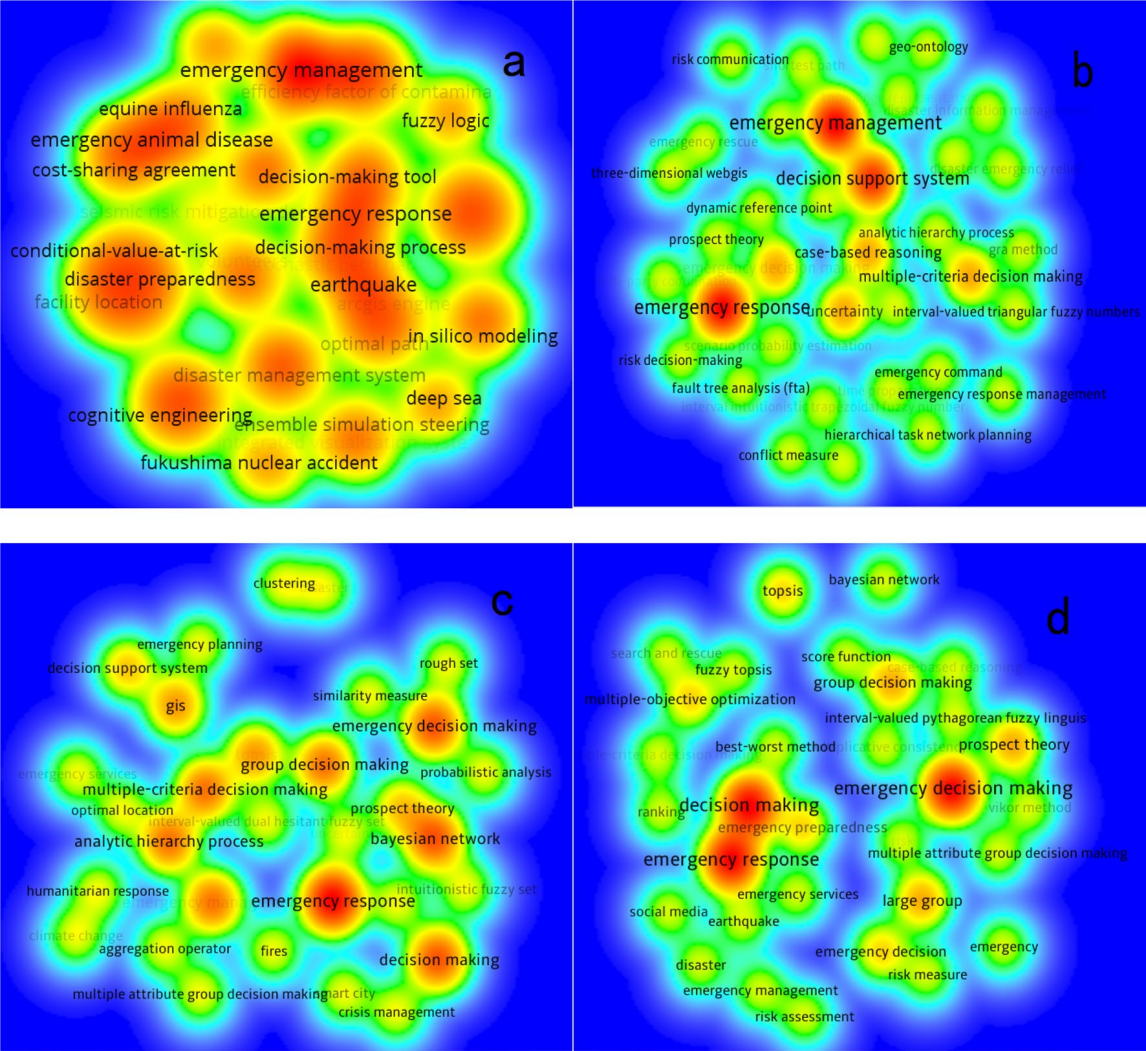
Hot terms of EDM keywords in different periods; **a** 2010–2012; **b** 2013–2015; **c** 2016–2018; **d** 2019–2020

Figure 8 shows the density view of keywords at different time periods, in which the color of a node lies on the occurrences of keyword associated with the node, where the frequency of keyword occurrences increases from blue to red color. There were 109 keywords in the first period (2010–2012) which could be regarded as the early stage of research in the EDM field. In this period, emergency management, emergency animal disease, earthquake, emergency response, and decision analysis are the major research themes. A total of 127 keywords appear in the second period (2013–2015). In this period, emergency management and emergency response are still hot topics. In addition, researchers have begun to focus more on decision support system, case-based reasoning, prospect theory, GRA method, and fault tree analysis to handle unconventional emergency events. There are 353 keywords in the third period (2016–2018). These keywords indicate that researches focused on emergency response, Bayesian network, emergency decision-making, emergency management, analytic hierarchy process, group decision-making, multiple-criteria decision-making, GIS, and TOPSIS. In addition, 368 keywords appear in the most recent period (2019–2020). Emergency response, emergency decision-making, prospect theory, and large group decision-making are the main topics.

**Fig. 9:**
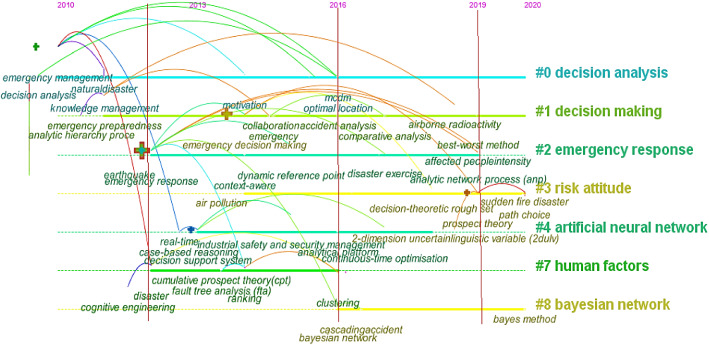
Keyword timeline analysis result

Figure 9 depicts the time line visualization of co-occurrence keywords network and associated clustering analysis result. According to this figure, the following results can be obtained: (1) The research period on EDM improvement can be divided into nascency stage, growth stage, explosion stage, and lucubration stage. The first 10 years (2010–2012) is the nascency stage, in which rare co-occurrence keywords emerged. Between 2012 and 2014, more researchers focused on the research about “air pollution”, “industrial safety and security management”, “case-based reasoning”, “cumulative prospect theory”, “fault tree analysis” and “decision support system”, and the studies of this field began to grow progressively. From 2014 to 2019, “MCDM”, “dynamic reference point” and “Bayesian network” have entered the major keywords spot in this period. In recent two years, from 2019 to 2020, the studies in this field have utilized more advanced and effective techniques like “analytic network process”, “best–worst method” and “2-dimension uncertain linguistic variable” to address EDM problems. (2) All the co-occurrence keywords are classified into seven communities with different labels. Two clusters #0 decision analysis and #1 decision-making are formed by the researches from 2010 until now. Between 2012 and 2016, four areas have attracted a lot of attention from researchers, which include #2 emergency response, #3 risk attitude and #4 artificial neural network. From 2016 to now, except for the clusters (#0, #1, and #3) that are constantly being concerned, another major cluster is #8 Bayesian network. Besides, In the clusters of #1 decision-making, #2 emergency response, #3 risk attitude, #4 artificial neural network, #7 human factors and #8 Bayesian network, many techniques have been proposed to overcome the flaws regarding EDM.

## EDM methods used in the literature

Through the analysis of the selected literature, the prospect theory is found to be the most popular method used in EDM, which can consider the decision-makers’ psychological behavior in decision analysis. In Ref. [35], a risk decision analysis method based on cumulative prospect theory (CPT) was proposed to handle the risk decision-making problem in emergency response. Ren et al. [41] introduced the negative exponential function into the prospect theory to deal with EDM problems. Wang et al. [53] suggested a prospect theory-based interval dynamic reference point method for EDM, Wang et al. [51] provided a group decision approach based on prospect theory for emergency situations, and Zhang et al. [76] introduced an EDM method based on prospect theory for different emergency situations. Hao et al. [26] developed a dynamic decision-making method integrating prospect theory to solve the mine EDM problems. Xu et al. [61] reported a risk-based dynamic EDM method based on the dual influence of preference transfer and cumulative prospect theory. Recently, Ding and Liu [13] introduced an integrated approach based on prospect theory and VIKOR for EDM with two-dimension uncertain linguistic information. Ding et al. [15] presented a dynamic approach for EDM based on prospect theory and interval-valued Pythagorean fuzzy linguistic variables. Xu et al. [59] put forward a multi-stage EDM method based on cumulative prospect theory and intuitionistic fuzzy numbers considering the decision-maker’s emotion update mechanism.

As an extension of the prospect theory, TODIM (an acronym in Portuguese of interactive and multiple attribute decision-making) was also frequently used method to handle EDM problems. Li and Cao [33] provided a risk decision analysis method based on the TODIM method, in which criteria values and state probabilities were expressed in interval numbers. Liang et al. [34] extended the TODIM method into multi-granularity proportional hesitant fuzzy linguistic environment for mine EDM. Ding et al. [10] applied an interval-valued hesitant fuzzy TODIM method to tackle the dynamic EDM problem, Ding et al. [12] a probabilistic hesitant fuzzy TODIM method for dynamic EDM, Ding et al. [11] proposed an extended TODIM approach for EDM based on bidirectional projection with hesitant triangular fuzzy sets.

In addition, the technique for order preference by similarity to ideal solution (TOPSIS) is a popular method to cope with the EDM problems, as it determines the most satisfactory alternative considering both positive ideal solution and the negative ideal solution. In this aspect, Zhang et al. [73] developed a fuzzy model for emergency management evaluation based on the idea of TOPSIS. Wang et al. [51] introduced a fuzzy TOPSIS method for dynamic EDM considering experts’ hesitation. Ju and Wang [29] proposed a method based on Dempster-Shafer theory and interval TOPSIS to solve EDM problems with incomplete information, and Ju et al. [30] presented a framework combining analytic network process (ANP), decision-making trial and evaluation laboratory (DEMATEL), and 2-tuple linguistic TOPSIS for emergency alternative evaluation and selection. In Ref. [45], an approach to evaluate the emergency plans for emergency events based on TOPSIS method and soft fuzzy rough set theory. In Ref. [58], a grey relational analysis (GRA)-based TOPSIS model was established to deal with the oil spill emergency management problems. In Ref. [2], a spherical fuzzy emergency decision support model was suggested by integrating GRA and TOPSIS methods. Besides, the Z-uncertain probabilistic linguistic TOPSIS [6], the intuitionistic fuzzy TOPSIS [7], the Pythagorean fuzzy TOPSIS [72], and the Probabilistic hesitant fuzzy TOPSIS [44] were proposed for emergency alternative evaluation in recent years.

## Challenges and future directions

Although EDM has made great progress, there are still many limitations and challenges. Many improvements in methods for timely and effective EDM are still needed to enrich this field. In this section, we discuss some current challenges and possible directions for further investigations.

### Current challenges

With the rapid propagation of information and the sharp expansion of globalization, EDM is becoming more complex and uncertain nowadays. To make more satisfactory and reliable decisions in the shortest possible time, the following challenges could be considered:

1. For the time starved in the emergency response, there exists much uncertain and vague decision-making information due to the evolution of emergency situations. Moreover, the information available for EDM is usually incomplete or even unknown, especially in the early stages. Therefore, the foremost issue in EDM is how to handle uncertainty and incomplete data in complex and dynamic emergency environment.
2. Decision-makers in the EDM often have different experience, backgrounds, and interests, and thus conflict opinions are unavoidable when eliciting assessments over emergency responses [59, 67]. Thus, another challenge to address an EDM problem is how to solve conflict judgements of decision-makers to improve group consistency and lead to efficiency improvement in the actual decision-making process.
3. To determine the most desirable emergency response, various EDM models and algorithms have been proposed in previous studies. But different approaches can yield different rankings and each one has its own advantages and disadvantages. Therefore, the key is how theoretical results can be reconciled with practical applications, approaching this reconciliation not from the theoretical results but from the real-world EDM problems.
4. In recent years, data has started to generate on a large volume in the EDM field. It has been expected that the amount of data will continue to increase largely due to the complexity of emergency events. Therefore, with regard to the presentation of big data, which is mostly unstructured text, it is a challenge to deal with such data in the EDM.

### Future directions

Based on the bibliometric analyses of EDM literature, we here suggest some future research directions to facilitate the development of EDM, and the following aspects may be interesting:

1. Till now, some uncertainty theories and methods, such as probabilistic linguistic term sets [64], hesitant triangular fuzzy sets [11], Pythagorean probabilistic hesitant fuzzy sets [3], Pythagorean fuzzy rough sets [72], picture fuzzy sets [16], spherical fuzzy sets [2], and Z-uncertain probabilistic linguistic variables [6], have been used to deal with the uncertain and vague information in EDM. Over time, the EDM problems have been more complex and required both quantitative and qualitative information. In further studies, more types of flexible fuzzy and linguistic expressions are supposed to be adopted to deal with complex multi-layer information in EDM. Besides, it is interesting to investigate the EDM methods that could handle multiple types of information in practice.
2. Group conflict is the main factor hindering the achievement of group consensus for EDM, especially in the large group environment. To approach this issue, many studies have utilized the consensus method [62–64], consistency index [21, 54, 60], and expert weighting method [5, 65] to reach a consensus or eliminate conflict in the EDM process. In the future, it would be interesting to consider decision-makers’ bounded confidence [75], knowledge structures [17], sub-group cohesiveness [42], opinions evolution [9], and dynamic trust [49] during the consensus reaching process in EDM.
3. Many researches have been conducted by extending the classic MCDM methods in EDM, such as the prospect theory [35, 51, 52], the TOPSIS [6, 45], the TODIM [33, 34], the analytic network process (ANP) [79], and the combinative distance-based assessment (CODAS) [39]. But the applied decision-making methods are still limited. Considering the effectiveness and practicability, some new and outstanding methods could be introduced and employed for EDM applications. For example, the evaluation based on distance from average solution (EDAS) method [22], the measuring attractiveness by a categorical-based evaluation technique (MACBETH) [19], and the alternative queuing method [24] are relatively new or popular MCDM methods, and they may improve the performance for EDM results.
4. Another possible direction for future work would be to apply artificial intelligence techniques to efficiently improve the capability and enhance the features of EDM. For example, deep learning-based optimization models [1, 20] can be used to calculate the weight vector of criteria weights objectively based on the assessment information of emergency schemes. Also, EDM methods can be empowered by neural networks to consider fluctuations in the determination of a desirable alternative when responding to an emergency event. Therefore, the EDM researches combined with artificial intelligence may be a hotspot research direction in the future.
5. The development of Internet of Things has led to commensurate developments in complex devices and technologies and the use of big data for comprehensive analyses. However, there have been few articles that have used big data to address EDM problems, most of which also have not included data mining. As big data analysis and data mining play a huge role in emergency forecasting, solution evaluations, and risk reduction, future researches need to apply data mining technologies to discover more features and underlying information and reform the EDM process.

Moreover, there are many other research directions in EDM, and the development of bibliometric analysis enables us to grasp hot topics and predict the trends of EDM from many aspects. For one thing, keywords in different periods could help scholars understand the development of EDM, and they can further obtain some new ideas after analysis. For another, when scholars need to communicate and cooperate, they could refer to prolific authors, institutions and countries in the EDM area. In a word, the bibliometric analysis conducted in this study provides valuable information for scholars to understand the EDM research field.

## Conclusions

In this article, we provided a comprehensive overview and clearly visualized analysis of the literature on EDM published between 2010 and 2020. A total of 303 journal articles were identified to carry on the bibliometric analysis and comprehensive review. From the trend of publications, the number of articles on EDM has substantially increased after 2014, and the majority of publications were published in the past five years. Via the cooperation network analysis, it is found that China dominates the research field based on the total papers and total citations; Turkey has the largest influence in EDM according to the average citations. Wuhan University of Technology is the most prolific institution in the EDM field according to the total publications; Sichuan University attracted the most attention from researchers in EDM according to the total citations. Xu Xuanhua is the most productive and important author according to the total publications and total citations and. Up to now, several main research teams have been formed, but the connection among them is weak and academic communications are few.

In terms of the co-citation network analysis, the most influential paper is Ref. [66] as it has been cited most by other documents in the EDM field. In addition, Refs. [35, 79], and Ref. [31] are the core articles of their respective research directions. The International Journal of Disaster Risk Reduction is the most publication source in EDM, and the articles published in the Knowledge-Based Systems are the most cited by the documents in the EDM field.

The keyword co-occurrence analysis showed that “emergency decision-making”, ‘‘emergency response”, “emergency management”, “multiple-criteria decision-making”, “Bayesian network”, “TOPSIS”, “decision-making support”, and “prospect theory” are the most frequently appeared keywords in the EDM literature. The “decision analysis”, “decision-making”, “risk attitude”, and “Bayesian network” are constantly being concerned in the field and will be potential research topics in the near future. To sum up, the present work is helpful for researchers practitioners interested in the EDM to gain a deeper understanding of current research hotspots and potential research directions.

## References

[1] Asghar MZ, Subhan F, Ahmad H, Khan WZ, Hakak S, Gadekallu TR, Alazab M (2021). Senti-eSystem: a sentiment-based eSystem-using hybridized fuzzy and deep neural network for measuring customer satisfaction. Softw Pract Exp.

[2] Ashraf S, Abdullah S (2020). Emergency decision support modeling for COVID-19 based on spherical fuzzy information. Int J Intell Syst.

[3] Batool B, Abosuliman SS, Abdullah S, Ashraf S (2021). EDAS method for decision support modeling under the Pythagorean probabilistic hesitant fuzzy aggregation information. J Ambient Intell Humaniz Comput.

[4] Bond S, Cooper S (2006). Modelling emergency decisions: recognition-primed decision-making. The literature in relation to an ophthalmic critical incident. J Clin Nurs.

[5] Cai CG, Xu XH, Wang P, Chen XH (2017). A multi-stage conflict style large group emergency decision-making method. Soft Comput.

[6] Chai J, Xian S, Lu S (2021) Z-uncertain probabilistic linguistic variables and its application in emergency decision-making for treatment of COVID-19 patients. Int J Intell Syst. 36(1):362–402

[7] Chen L, Li Z, Deng X (2020). Emergency alternative evaluation under group decision-makers: a new method based on entropy weight and DEMATEL. Int J Syst Sci.

[8] Chen N, Liu W, Bai R, Chen A (2019). Application of computational intelligence technologies in emergency management: a literature review. Artif Intell Rev.

[9] Chen X, Ding Z, Dong Y, Liang H (2021). Managing consensus with minimum adjustments in group decision-making with opinions evolution. IEEE Trans Syst Man Cybern Syst.

[10] Ding Q, Goh M, Wang YM (2021) Interval-valued hesitant fuzzy TODIM method for dynamic emergency responses. Soft Comput. 25:8263–8279 10.1007/s00500-021-05751-z PMC8076885 33935587

[11] Ding Q, Wang YM, Goh M (2021). An extended TODIM approach for group emergency decision-making based on bidirectional projection with hesitant triangular fuzzy sets. Comput Ind Eng.

[12] Ding Q, Wang YM, Goh M (2021) TODIM dynamic emergency decision-making method based on hybrid weighted distance under probabilistic hesitant fuzzy information. Int J Fuzzy Syst. 23:474–491

[13] Ding XF, Liu HC (2019). An extended prospect theory–VIKOR approach for emergency decision-making with 2-dimension uncertain linguistic information. Soft Comput.

[14] Ding XF, Liu HC (2019). A new approach for emergency decision-making based on zero-sum game with Pythagorean fuzzy uncertain linguistic variables. Int J Intell Syst.

[15] Ding XF, Liu HC, Shi H (2019). A dynamic approach for emergency decision-making based on prospect theory with interval-valued Pythagorean fuzzy linguistic variables. Comput Ind Eng.

[16] Ding XF, Zhang L, Liu HC (2020) Emergency decision-making with extended axiomatic design approach under picture fuzzy environment. Expert Syst. 37(2):e12482

[17] Du YW, Chen Q, Sun YL, Li CH (2021). Knowledge structure-based consensus-reaching method for large-scale multiattribute group decision-making. Knowl Based Syst.

[18] Fang C, Zhang J (2018). Performance of green supply chain management: a systematic review and meta analysis. J Clean Prod.

[19] Ferreira FAF, Santos SP (2021) Two decades on the MACBETH approach: a bibliometric analysis. Ann Oper Res. 296:901–925

[20] Gadekallu TR, Alazab M, Kaluri R, Maddikunta PKR, Bhattacharya S, Lakshmanna K, Parimala M (2021). Hand gesture classification using a novel CNN-crow search algorithm. Complex Intell Syst.

[21] Gao J, Xu Z, Liang Z, Liao H (2019). Expected consistency-based emergency decision-making with incomplete probabilistic linguistic preference relations. Knowl Based Syst.

[22] Ghorabaee MK, Zavadskas EK, Olfat L, Turskis Z (2015). Multi-criteria inventory classification using a new method of evaluation based on distance from average solution (EDAS). Informatica.

[23] Gil M, Wróbel K, Montewka J, Goerlandt F (2020). A bibliometric analysis and systematic review of shipboard Decision Support Systems for accident prevention. Safety Sci.

[24] Gou X, Xu Z, Liao H (2016). Alternative queuing method for multiple criteria decision-making with hybrid fuzzy and ranking information. Inf Sci.

[25] Han Y, Deng Y (2018). An enhanced fuzzy evidential DEMATEL method with its application to identify critical success factors. Soft Comput.

[26] Hao Z, Xu Z, Zhao H, Fujita H (2018). A dynamic weight determination approach based on the intuitionistic fuzzy Bayesian network and its application to emergency decision-making. IEEE Trans Fuzzy Syst.

[27] Hou L-X, Liu R, Liu H-C, Jiang S (2021). Two decades on human reliability analysis: a bibliometric analysis and literature review. Ann Nucl Energy.

[28] Huang J, You J-X, Liu H-C, Song M-S (2020). Failure mode and effect analysis improvement: a systematic literature review and future research agenda. Reliab Eng Syst Saf.

[29] Ju Y, Wang A (2012). Emergency alternative evaluation under group decision-makers: a method of incorporating DS/AHP with extended TOPSIS. Expert Syst Appl.

[30] Ju Y, Wang A, You T (2015). Emergency alternative evaluation and selection based on ANP, DEMATEL, and TL-TOPSIS. Nat Hazards.

[31] Kahneman D, Tversky A (1979). Prospect theory: an analysis of decision under risk. Econometrica.

[32] Khaldi H, Prado-Gascó V (2021). Bibliometric maps and co-word analysis of the literature on international cooperation on migration. Qual Quant.

[33] Li MY, Cao PP (2019). Extended TODIM method for multi-attribute risk decision-making problems in emergency response. Comput Ind Eng.

[34] Liang Y, Tu Y, Ju Y, Shen W (2019). A multi-granularity proportional hesitant fuzzy linguistic TODIM method and its application to emergency decision-making. Int J Disaster Risk Reduct.

[35] Liu Y, Fan ZP, Zhang Y (2014). Risk decision analysis in emergency response: a method based on cumulative prospect theory. Comput Oper Res.

[36] Molassiotis A, Guo C, Abu-Odah H, West C, Loke AY (2021). Evolution of disaster nursing research in the past 30 years (1990–2019): A bibliometric and mapping analysis. Int J Disaster Risk Reduct.

[37] Noyan N (2012). Risk-averse two-stage stochastic programming with an application to disaster management. Comput Oper Res.

[38] Palácios H, de Almeida MH, Sousa MJ (2021). A bibliometric analysis of trust in the field of hospitality and tourism. Int J Hospital Manag.

[39] Peng X, Garg H (2018). Algorithms for interval-valued fuzzy soft sets in emergency decision-making based on WDBA and CODAS with new information measure. Comput Ind Eng.

[40] Radcliffe K, Lyson HC, Barr-Walker J, Sarkar U (2019). Collective intelligence in medical decision-making: a systematic scoping review. BMC Med Inform Decis Mak.

[41] Ren P, Xu Z, Hao Z (2017). Hesitant fuzzy thermodynamic method for emergency decision-making based on prospect theory. IEEE Trans Cybern.

[42] Rodríguez RM, Labella Á, Sesma-Sara M, Bustince H, Martínez L (2021). A cohesion-driven consensus reaching process for large scale group decision-making under a hesitant fuzzy linguistic term sets environment. Comput Ind Eng.

[43] Rundo L, Pirrone R, Vitabile S, Sala E, Gambino O (2020). Recent advances of HCI in decision-making tasks for optimized clinical workflows and precision medicine. J Biomed Inf.

[44] Sha X, Yin C, Xu Z, Zhang S (2021). Probabilistic hesitant fuzzy TOPSIS emergency decision-making method based on the cumulative prospect theory. J Intell Fuzzy Syst.

[45] Sun B, Ma W (2016). An approach to evaluation of emergency plans for unconventional emergency events based on soft fuzzy rough set. Kybernetes.

[46] Sun B, Ma W, Zhao H (2016). An approach to emergency decision-making based on decision-theoretic rough set over two universes. Soft Comput.

[47] Sun B, Ma W, Li B, Li X (2018) Three-way decisions approach to multiple attribute group decision making with linguistic information-based decision-theoretic rough fuzzy set. Int J Approx Reason 93:424–442.

[48] Tan X, Zhu J, Cabrerizo FJ, Herrera-Viedma E (2021). A cyclic dynamic trust-based consensus model for large-scale group decision-making with probabilistic linguistic information. Appl Soft Comput.

[49] Tandon A, Kaur P, Mäntymäki M, Dhir A (2021). Blockchain applications in management: a bibliometric analysis and literature review. Technol Forecast Soc Change.

[50] Wang L, Rodríguez RM, Wang YM (2018). A dynamic multi-attribute group emergency decision-making method considering experts’ hesitation. Int J Comput Intell Syst.

[51] Wang L, Wang YM, Martínez L (2017). A group decision method based on prospect theory for emergency situations. Inf Sci.

[52] Wang L, Zhang ZX, Wang YM (2015). A prospect theory-based interval dynamic reference point method for emergency decision-making. Expert Syst Appl.

[53] Wen QJ, Ren ZJ, Lu H, Wu JF (2021). The progress and trend of BIM research: a bibliometrics-based visualization analysis. Autom Constr.

[54] Wu W, Peng Y (2016). Extension of grey relational analysis for facilitating group consensus to oil spill emergency management. Ann Oper Res.

[55] Wu B, Yan XP, Wang Y, Zhang D, Soares CG (2017a) Three-Stage decision-making model under restricted conditions for emergency response to ships not under control. Risk Anal 37:2455–2474. 10.1111/risa.12815 28437861

[56] Wu B, Zong L, Yan X, Guedes Soares C (2018). Incorporating evidential reasoning and TOPSIS into group decision-making under uncertainty for handling ship without command. Ocean Eng.

[57] Wu Q, Wu P, Zhou L, Chen H, Guan X (2018). Some new Hamacher aggregation operators under single-valued neutrosophic 2-tuple linguistic environment and their applications to multi-attribute group decision-making. Comput Ind Eng.

[58] Wu B, Zhao C, Yip TL, Jiang D (2021). A novel emergency decision-making model for collision accidents in the Yangtze River. Ocean Eng.

[59] Xu J, Guo J, Zhang J, Liu W, Ma H (2021). Multi-stage emergency decision-making method based on cumulative prospect theory and intuitionistic fuzzy number. RAIRO Oper Res.

[60] Xu X, Huang Y, Chen K (2019). Method for large group emergency decision-making with complex preferences based on emergency similarity and interval consistency. Nat Hazards.

[61] Xu X, Pan B, Yang Y (2018). Large-group risk dynamic emergency decision method based on the dual influence of preference transfer and risk preference. Soft Comput.

[62] Xu X, Zhang Q, Chen X (2020). Consensus-based non-cooperative behaviors management in large-group emergency decision-making considering experts’ trust relations and preference risks. Knowl Based Syst.

[63] Xu XH, Du ZJ, Chen XH (2015). Consensus model for multi-criteria large-group emergency decision-making considering non-cooperative behaviors and minority opinions. Decis Support Syst.

[64] Xu XH, Zhong XY, Chen XH, Zhou YJ (2015). A dynamical consensus method based on exit-delegation mechanism for large group emergency decision-making. Knowl Based Syst.

[65] Xu Y, Zhang W, Wang H (2015). A conflict-eliminating approach for emergency group decision of unconventional incidents. Knowl Based Syst.

[66] Xue W, Xu Z, Mi X, Ren Z (2021). Dynamic reference point method with probabilistic linguistic information based on the regret theory for public health emergency decision-making. Economic Research-Ekonomska Istrazivanja.

[67] Yaghoubi T, Ardalan A, Zavareh DK, Khankeh H, Nejati A, Ebadi A (2017). Decision-making on hospital emergency evacuation in disasters and emergencies: findings from a systematic review. Iranian Red Crescent Med J.

[68] Yates D, Paquette S (2011). Emergency knowledge management and social media technologies: a case study of the 2010 Haitian earthquake. Int J Inf Manage.

[69] Yin X, Xu X, Chen X (2020). Risk mechanisms of large group emergency decision-making based on multi-agent simulation. Nat Hazards.

[70] Yu F, Li X-Y (2018). Improving emergency response to cascading disasters: applying case-based reasoning towards urban critical infrastructure. Int J Disaster Risk Reduct.

[71] Zadeh LA (1975). The concept of a linguistic variable and its application to approximate reasoning—I. Inf Sci.

[72] Zhan J, Sun B, Zhang X (2020). PF-TOPSIS method based on CPFRS models: an application to unconventional emergency events. Comput Ind Eng.

[73] Zhang G, Ma J, Lu J (2009). Emergency management evaluation by a fuzzy multi-criteria group decision support system. Stoch Env Res Risk Assess.

[74] Zhang L, Wang YZ, Zhao XY (2018). A new emergency decision support methodology based on multi-source knowledge in 2-tuple linguistic model. Knowl Based Syst.

[75] Zhang Z, Li Z, Gao Y (2021). Consensus reaching for group decision-making with multi-granular unbalanced linguistic information: a bounded confidence and minimum adjustment-based approach. Inf Fusion.

[76] Zhang ZX, Wang L, Wang YM (2018). An emergency decision-making method based on prospect theory for different emergency situations. Int J Disaster Risk Sci.

[77] Zhao X, Ke Y, Zuo J, Xiong W, Wu P (2020). Evaluation of sustainable transport research in 2000–2019. J Clean Product.

[78] Zhou L, Wu X, Xu Z, Fujita H (2018). Emergency decision-making for natural disasters: an overview. Int J Disaster Risk Reduct.

[79] Zhou X, Wang L, Qin J, Chai J, Gómez Muñoz CQ (2019). Emergency rescue planning under probabilistic linguistic information: an integrated FTA-ANP method. Int J Disaster Risk Reduct.

